# Epitope Addition and Ablation via Manipulation of a Dengue Virus Serotype 1 Infectious Clone

**DOI:** 10.1128/mSphere.00380-16

**Published:** 2017-02-22

**Authors:** Emily N. Gallichotte, Vineet D. Menachery, Boyd L. Yount, Douglas G. Widman, Kenneth H. Dinnon, Steven Hartman, Aravinda M. de Silva, Ralph S. Baric

**Affiliations:** a Department of Microbiology and Immunology, University of North Carolina School of Medicine, Chapel Hill, North Carolina, USA; b Department of Epidemiology, Gillings School of Global Public Health, University of North Carolina, Chapel Hill, North Carolina, USA; University of Kentucky College of Medicine

**Keywords:** antibody, dengue, epitope, infectious clone

## Abstract

Despite the clinical relevance, dengue virus (DENV) research has been hampered by the absence of robust reverse genetic systems to manipulate the viral serotypes for propagation and generation of mutant viruses. In this article, we describe application of an infectious clone system for DENV serotype 1 (DENV1). Similar to previous clones in both flaviviruses and coronaviruses, the approach constructs a panel of contiguous cDNAs that span the DENV genome and can be systematically and directionally assembled to produce viable, full-length viruses. Comparison of the virus derived from the infectious clone with the original viral isolate reveals identical sequence, comparable endpoint titers, and similar focus staining. Both focus-forming assays and percent infection by flow cytometry revealed overlapping replication levels in two different cell types. Moreover, serotype-specific monoclonal antibodies (MAbs) bound similarly to infectious clone and the natural isolate. Using the clone, we were able to insert a DENV4 type-specific epitope recognized by primate MAb 5H2 into envelope (E) protein domain I (EDI) of DENV1 and recover a viable chimeric recombinant virus. The recombinant DENV1 virus was recognized and neutralized by the DENV4 type-specific 5H2 MAb. The introduction of the 5H2 epitope ablated two epitopes on DENV1 EDI recognized by human MAbs (1F4 and 14C10) that strongly neutralize DENV1. Together, the work demonstrates the utility of the infectious clone and provides a resource to rapidly manipulate the DENV1 serotype for generation of recombinant and mutant viruses.

**IMPORTANCE** Dengue viruses (DENVs) are significant mosquito-transmitted pathogens that cause widespread infection and can lead to severe infection and complications. Here we further characterize a novel and robust DENV serotype 1 (DENV1) infectious clone system that can be used to support basic and applied research. We demonstrate how the system can be used to probe the antigenic relationships between strains by creating viable recombinant viruses that display or lack major antibody epitopes. The DENV1 clone system and recombinant viruses can be used to analyze existing vaccine immune responses and inform second-generation bivalent vaccine designs.

## Abstract (precis)

Dengue viruses (DENVs) are significant mosquito-transmitted pathogens that cause widespread infection and can lead to severe infection and complications. Here we further characterize a novel and robust DENV serotype 1 (DENV1) infectious clone system that can be used to support basic and applied research. We demonstrate how the system can be used to probe the antigenic relationships between strains by creating viable recombinant viruses that display or lack major antibody epitopes. The DENV1 clone system and recombinant viruses can be used to analyze existing vaccine immune responses and inform second-generation bivalent vaccine designs.

## INTRODUCTION

Dengue viruses (DENVs) represent a significant threat to human health and the global economy, causing widespread and frequent epidemics (1, 2). Transmitted by female mosquitos, the DENV family has four known serotypes that are genetically and antigenically related. Infection with any one DENV serotype typically produces asymptomatic or mild disease but induces long-term immunity against that serotype (3). However, subsequent exposure to a different serotype may result in more-severe disease characterized by dengue hemorrhagic fever or dengue shock syndrome often attributed to antibody-dependent enhancement (2, 4). Overall, potential adverse disease outcomes due to immune cross-reactivity complicates efforts to produce effective vaccines against the DENV family (5).

To study aspects of infection and pathogenesis, a number of reverse genetic systems have been generated for members of the flavivirus family, which includes DENV (6, 7). These approaches have provided powerful tools to manipulate the viral genome and have given insight into viral protein function, virulence determinants, viral immune evasion, host specificity, and other virus-host interactions (6). Reverse genetic systems have also facilitated improved research methodologies with the development of reporter assays as well as the means to produce virus-like particles and live attenuated vaccines (8, 9). In addition, infectious clones provide the opportunity to explore a clonal population of DENV with relatively few mutations versus the “mutant spectrum” typically observed in laboratory passaged stocks (10–12). Together, these tools have been critical in building a foundation to understanding DENV disease and pathogenesis.

Yet, while current systems have been useful, several obstacles remain for DENV reverse genetic systems (6). Flavivirus clones are difficult to maintain in bacteria and often result in sequence rearrangements or mutations that reduce expression of toxic elements from the viral genome. Several approaches have been employed to improve propagation, including use of low-copy-number plasmid (6), insertion into bacterial or yeast artificial chromosomes (13–15), disruption of promoter regions in viral cDNA (16), as well as microbe-free propagation approaches (17, 18). However, these methods often diminish yields, produce changes in viral sequence, or require significant extraneous effort that reduces the overall utility of the approaches. Importantly, many of these reverse genetic systems report significant attenuation relative to their control wild-type (WT) viruses despite similar consensus sequencing (6). Overall, these results highlight the obstacles for using reverse genetic systems to study DENV disease and pathogenesis.

In this study, we extend our characterization of a dengue virus serotype 1 (DENV1) infectious clone (19) by comparing and contrasting the growth and antigenic properties of molecularly cloned recombinant and wild-type DENV1. Importantly, DENV1 derived from the clone maintained equivalent replication and complete antibody binding fidelity with the wild-type strain. Using the tetrapartite DENV1 molecular clone, we then inserted a known DENV4 envelope protein domain I (EDI) neutralization epitope and demonstrated efficient gain of neutralization function. However, by introducing these changes, we simultaneously ablated two known DENV1 antibody binding sites and disrupted their binding and neutralization. Together, the results illustrate the utility of this newly developed infectious clone as a platform to study DENV immunity by exchanging functional complex epitopes between DENV strains, simultaneously gaining new neutralization properties to other strains while ablating the primary neutralizing antigenic sites of DENV1.

## RESULTS

### Design and construction of DENV1 full-length infectious clone.

**FIG 1:**
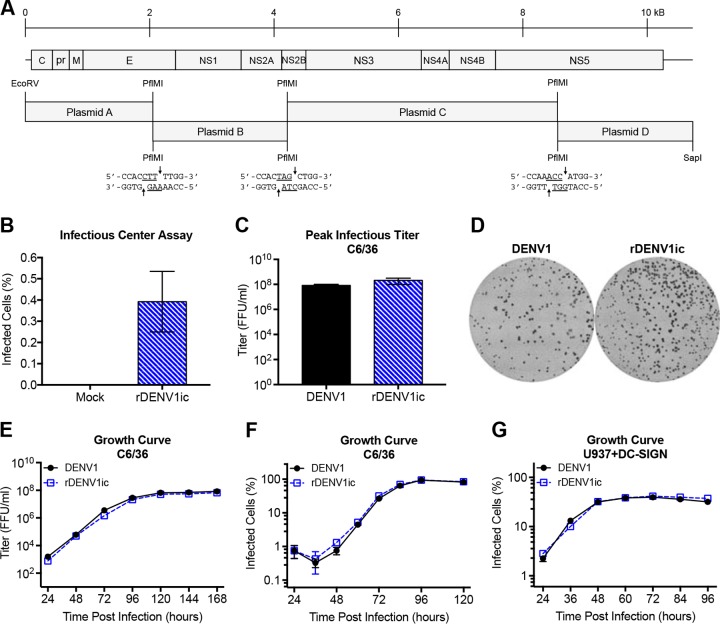
DENV1 infectious clone design, characterization, and growth. (A) Structure of DENV1 viral genome and division into the four subgenomic fragments designated plasmids A (nucleotides [nt] 1 to 2052), B (nt 2053 to 4215), C (nt 4216 to 8563), and D (nt 8564 to 10736). (B) Full-length viral RNA was electroporated into C6/36 cells, and the cells were diluted and added to confluent C6/36 cells. After incubation, infected cells were stained to determine the initial percentage of electroporated cells capable of producing infectious virus. (C) Peak infectious titers of virus stocks were determined on C6/36 cells. The titers of virus were determined in focus-forming units (FFU) per milliliter. (D) Immunostaining of DENV1 and rDENV1ic foci in C6/36 cells. (E) C6/36 cells were inoculated at an MOI of 0.01, and every 24 h, supernatant containing virus was harvested, and the titers of the virus were subsequently determined on C6/36 cells. (F and G) C6/36 cells were inoculated at an MOI of 0.01 (F) or 1% of DC-SIGN-expressing U937 (U937+DC-SIGN) cells were infected (G), and every 12 h, the cells were harvested and stained for intracellular viral antigen, and the total percentage of infected cells was calculated.

The recombinant DENV1 infectious clone (rDENV1ic) was synthesized as a panel of four continuous cDNA segments that span the entire genome (Fig. 1A) (19). Based on sequence from a laboratory strain of DENV1 West Pac ‘74, each segment is flanked by class IIS restriction endonuclease sites that cleave palindromic sequences into asymmetric 3-nucleotide overhangs. These overhangs permit directional assembly of the cleaved fragments to generate full-length viral cDNA. With the exception of an ablated restriction site (see Materials and Methods), the sequence directly reconstitutes the wild-type (WT) laboratory strain. The ligated cDNA fragments are then used as a template to produce full-length viral RNA, which is electroporated into C6/36 mosquito cells. To demonstrate transfection efficiencies of full-length transcripts, the results of the infectious center assay indicate low, but stable infection (0.4%) (Fig. 1B). After several days, cell culture supernatant containing progeny rDENV1ic virions is harvested and passaged once on C6/36 cells to generate working virus stocks.

RNA isolated from infected cells was verified relative to the parental wild-type strain using genome length analysis and finding no differences in the consensus amino acid sequences. Similarly, endpoint titers from C6/36 cells (Fig. 1C) found no notable difference between the viruses derived from wild-type DENV1 and rDENV1ic in regard to replication. In C6/36 cells, both wild-type DENV1- and infectious-clone-derived virus maintain similar sized foci following infection (Fig. 1D). Together, the results indicate that no replication deficit nor focus formation deficit was observed between the wild-type and infectious clone virus.

### Replication kinetics of rDENV1ic.

Some of the previously described reverse genetic systems for DENVs have been hampered by replication attenuation relative to the wild-type strain despite identical consensus sequences (6). To determine whether rDENV1ic is attenuated relative to the wild-type virus, growth curves were completed in C6/36 mosquito cells over a 7-day time course (Fig. 1E). The data indicate that wild-type DENV1 and rDENV1ic replicate equivalently, peaking in focus-forming units 5 days after infection (Fig. 1E). Percent infection of C6/36 cells also demonstrated no significant difference between the wild-type and infectious clone with nearly 100% infection observed 4 days after infection (Fig. 1F). We next examined replication in the human monocyte-derived U937 cell line stably expressing DC-SIGN (dendritic cell-specific intercellular adhesion molecule 3-grabbing nonintegrin), a known DENV attachment factor (Fig. 1G); similar to the C6/36 mosquito cell line, we observed no difference in the replication of the wild-type DENV1 and rDENV1ic in DC-SIGN-expressing U937 (U937+DC-SIGN) cells. These results demonstrate no detectable attenuation of the rDENV1ic clone relative to the wild-type strain in either insect or human cells.

### Display of antibody epitopes on rDENV1ic.

**FIG 2:**
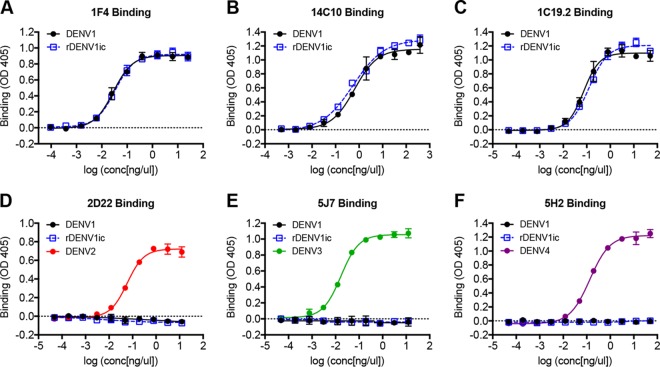
rDENV1ic is bound only by DENV1 serotype-specific antibodies. DENV1 and rDENV1ic were analyzed for their ability to bind DENV1 serotype-specific antibodies 1F4, 14C10, and 1C19.2 (A to C) or DENV2, DENV3, and DENV4 serotype-specific antibodies 2D22, 5J7, and 5H2 (D to F) in an ELISA virus capture assay. Binding measured by the optical density at 405 nm (OD 405) is shown on the *y* axes, and the log concentration (in nanograms per microliter) is shown on the *x* axes.

Antibody binding and neutralization are critical issues in DENV pathogenesis in humans; as such, the infectious clone and parental WT strain must display the same surface architecture and epitopes. To compare the display of antibody epitopes, enzyme-linked immunosorbent assays (ELISAs) were performed using a panel of DENV1-specific monoclonal antibodies (MAbs) against rDENV1ic and the parental wild-type strain (see Table S1 in the supplemental material). MAb 1F4, derived from a DENV1-infected patient (20), binds in the envelope domain I (EDI) region and extends into the EDI and domain II (EDII) hinge region (21); ELISA with MAb 1F4 produced strong and equivalent binding to both the wild-type and infectious clone (Fig. 2A). Similarly, the EDI, EDIII, and EDI/II hinge region is the epitope for MAb 14C10, which also bound similarly between the wild-type DENV1 and rDENV1ic (Fig. 2B). Finally, MAb 1C19.2, an EDIII antibody, also bound similarly to both wild-type DENV1 and rDENV1ic (Fig. 2C). Together, the results indicate that rDENV1ic displays the same antibody epitopes as the wild-type control virus.

10.1128/mSphere.00380-16.1 TABLE S1  Monoclonal antibody serotype specificity and epitopes. The serotype specificity, species, and epitopes of MAbs used in experiments are shown. Download TABLE S1, PDF file, 0.04 MB. Copyright © 2017 Gallichotte et al. 2017 Gallichotte et al. This content is distributed under the terms of the Creative Commons Attribution 4.0 International license.

We also tested the binding of DENV serotype 2, 3, and 4 monoclonal antibodies (Table S1) to rDENV1ic. Beginning with a DENV2-specific antibody, MAb 2D22 (22), failed to bind to either wild-type DENV1 or rDENV1ic (Fig. 2D). Similarly, MAb 5J7 (23) also fails to bind to rDENV1ic or wild-type strain while efficiently binding DENV3 (Fig. 2E). Finally, MAb 5H2, an EDI antibody specific to DENV4, showed no binding to either DENV1 iteration (Fig. 2F). Together, combined with DENV1 monoclonal data, the results indicate that rDENV1ic has the same surface architecture and epitopes as the wild-type strain.

### Antibody neutralization of rDENV1ic.

**FIG 3:**
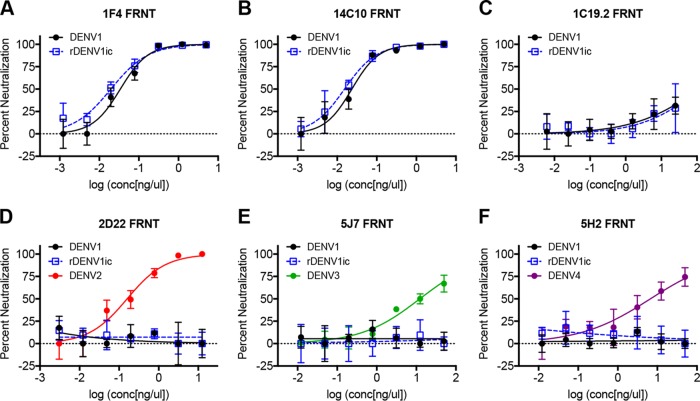
rDENV1ic maintains fidelity to DENV1 serotype-specific antibodies in a focus reduction neutralization test. Neutralization of DENV1 and rDENV1ic by monoclonal antibodies was measured in a C6/36 focus reduction neutralization test (FRNT) using DENV1 serotype-specific MAbs 1F4, 14C10, and 1C19.2 (A to C) and DENV2, DENV3, and DENV4 serotype-specific MAbs 2D22, 5J7, and 5H2 (D to F).

While observing equivalent antibody binding between rDENV1ic and wild-type virus by ELISA, neutralization is an even more sensitive functional assay to compare viruses. Small changes in binding conformation may induce enhancement versus neutralization; as such, these assays must be equivalent between the infectious clone and the wild-type virus to justify the use of rDENVic for vaccine and immunogenicity studies. Using a focus reduction neutralization test (FRNT), we examined the ability of well-defined MAbs to block virus infectivity. For all three DENV1 monoclonal antibodies (1F4, 14C10, and 1C19.2), percent neutralization was nearly identical between the wild-type DENV1 and rDENV1ic (Fig. 3A to C). Similar to ELISA results, monoclonal antibodies against the other DENV serotypes were unable to neutralize either the clone or wild-type virus (Fig. 3D to F). Extending studies to a U937+DC-SIGN flow-based neutralization assay, percent infection results revealed overlapping neutralization of rDENV1ic and wild-type DENV1 following DENV1 monoclonal antibody incubations (Fig. S1A to C). In addition, none of the other DENV serotype-specific antibodies provides any significant reduction in viral infection (Fig. S1D to F). Together, the results match the ELISA data and indicate antibody neutralization fidelity between the infectious clone and wild-type viruses in two independent neutralization assays.

10.1128/mSphere.00380-16.2 FIG S1  rDENV1ic is neutralized only by DENV1 serotype-specific antibodies in a focus reduction neutralization test. Neutralization of DENV1 and rDENV1ic by monoclonal antibodies was measured in U937+DC-SIGN flow cytometry neutralization assay using DENV1 serotype-specific MAbs 1F4, 14C10, and 1C19.2 (A to C) and DENV2, DENV3, and DENV4 serotype-specific antibodies 2D22, 5J7, and 5H2 (D to F). Download FIG S1, PDF file, 0.2 MB. Copyright © 2017 Gallichotte et al. 2017 Gallichotte et al. This content is distributed under the terms of the Creative Commons Attribution 4.0 International license.

### Gain of DENV4 monoclonal binding and neutralization.

**FIG 4:**
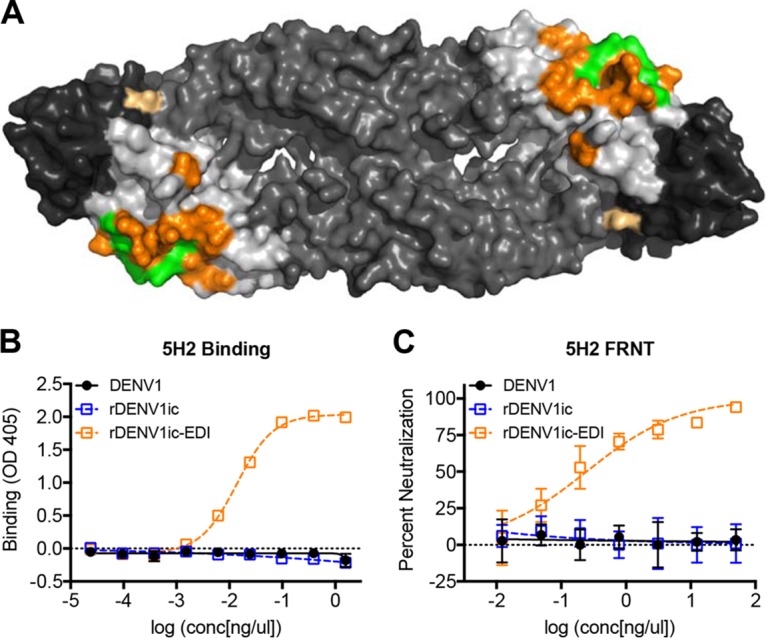
rDENV1ic-EDI gains binding and neutralization to DENV4 serotype-specific MAb 5H2. (A) Structure of DENV envelope protein dimer showing transplanted residues from DENV4 (orange) and MAb 5H2 epitope contact residues (green). Residues that are part of both the 5H2 MAb epitope and rDENV1ic-EDI virus are shown in orange. rDENV1ic-EDI virus that was recovered contained an additional mutation (light orange) unrelated to the transplanted 5H2 epitope. (B) Viruses were analyzed in an ELISA virus capture assay for their ability to bind DENV4-specific MAb 5H2. (C) Neutralization of viruses in C6/36 focus reduction neutralization test (FRNT) with DENV4-specific MAb 5H2.

To use the rDENVic to study antibody responses to specific epitopes, we generated a DENV1 mutant virus that incorporates the DENV4 5H2 epitope into the DENV1 backbone virus. 5H2 is a DENV4 type-specific and strongly neutralizing MAb isolated from a nonhuman primate infected with DENV4 (24, 25). A high-resolution structure of 5H2 bound to DENV4 E protein has led to the identification of 17 residues within EDI that interact with the antibody (Table 1) (24, 25). Of these 17 amino acids, five are conserved between DENV1 and DENV4. The remaining 12 residues in DENV1 were replaced with those from DENV4, by manipulating plasmid A of the DENV1 infectious clone (rDENV1ic-5H2-epitope [rDENV1ic-EDI]) (Fig. 4A) (Table 1). The DENV4 MAb 5H2 bound and neutralized the rDENV1ic-EDI virus (Fig. 4B and C). As the parental clone and wild-type DENV1 were not recognized by 5H2 (Fig. 4B and C), these results confirm successful transfer of a conformational epitope from DENV4 into DENV1.

**TABLE 1:**
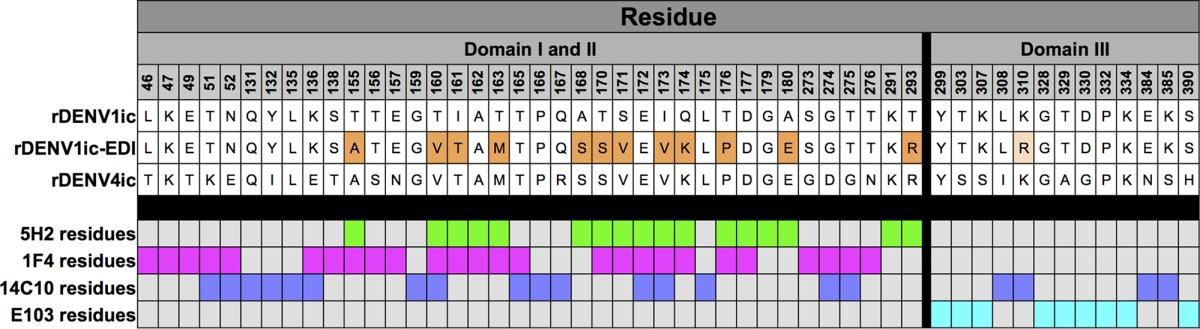
Virus amino acid sequences and MAb epitopes^a^

a Amino acid sequences for wild-type rDENV1ic, rDENV4ic are shown with chimeric rDENV1ic-EDI residues highlighted in orange (DENV4 amino acids transplanted into the DENV1 backbone sequence). rDENV1ic-EDI virus that was recovered contained an additional mutation (light orange) unrelated to the transplanted 5H2 epitope. Contact residues for MAbs 5H2, 1F4, 14C10, and E103 are shown in green, magenta, purple, and cyan, respectively.

### Ablation of DENV1 monoclonal binding and neutralization.

**FIG 5:**
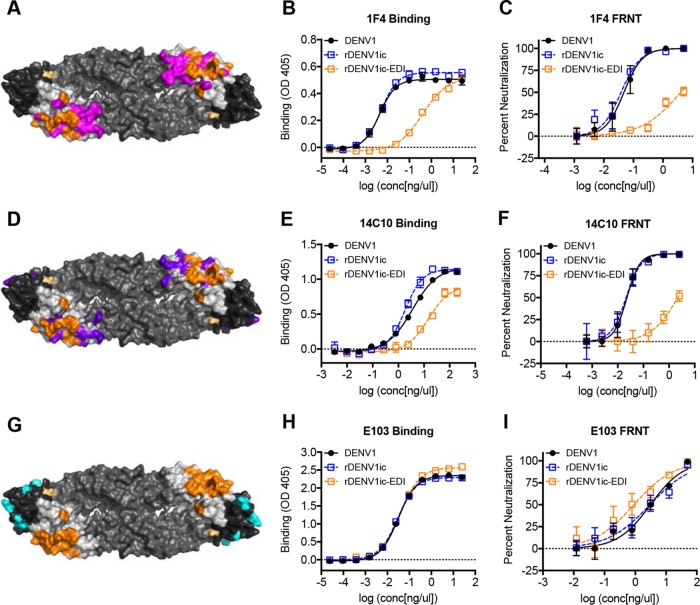
rDENV1ic-EDI loses binding and neutralization to EDI DENV1 serotype-specific MAbs. (A, D, and G) Structure of DENV envelope protein dimer showing transplanted residues from DENV4 (orange) and MAb epitope contact residues for 1F4 (magenta) (A), 14C10 (purple) (D), and E103 (cyan) (G). Residues that are part of both the MAb epitope and rDENV1ic-EDI virus are shown in orange. rDENV1ic-EDI virus that was recovered contained an additional mutation (light orange) unrelated to the transplanted 5H2 epitope. (B, E, and H) Viruses were analyzed in an ELISA virus capture assay for their ability to bind MAbs 1F4, 14C10, and E103. (C, F, and I) Neutralization of viruses in C6/36 focus reduction neutralization test (FRNT) with MAbs 1F4, 14C10, and E103.

The epitope of DENV4 MAb 5H2 overlaps with the known DENV1 type-specific epitopes recognized by human neutralizing MAbs 1F4 and 14C10 (21). Importantly, 1F4 has more than one-third of its predicted contact residues replaced in the rDENV1ic-EDI virus (Fig. 5A and Table 1). Insertion of the DENV4 5H2 epitope partially disrupted the DENV1 1F4 epitope, resulting in an 80-fold reduction in 50% effective concentration (EC_50_) but not complete ablation in binding of 1F4 to rDENV1ic-EDI (Fig. 5B). Similarly, neutralization assays indicate that 1F4 nearly lost all of its ability to neutralize the rDENV1ic-EDI mutant (Fig. 5C). MAb 14C10 has a smaller percentage (15%) of its epitope disrupted in the rDENV1ic-EDI virus (Fig. 5D and Table 1), as it has additional contact residues in EDIII from the neighboring dimer that are maintained (Fig. S2) (26). Despite this smaller percentage, binding of 14C10 is still reduced (Fig. 5E), and 14C10 neutralization of rDENV1ic-EDI is mostly lost (Fig. 5F). In contrast, DENV1 monoclonal antibody E103, which targets an EDIII epitope (Fig. 5G) (27), had no significant reduction in ELISA binding relative to wild-type virus or the infectious clone (Fig. 5H). Additionally, MAb E103 maintained robust neutralization, indicating maintenance of other DENV1 epitopes within the mutant virus (Fig. 5I). In summary, the introduction of the 5H2 epitope residues to create the rDENV1ic-EDI virus leads to efficient display of a heterologous DENV4 epitope and the disruption of two native DENV1 type-specific epitopes. Our results illustrate the utility of the infectious clone for transferring DENV epitopes between serotypes and identify functional consequences on other conserved epitopes within the transferred amino acid regions.

10.1128/mSphere.00380-16.3 FIG S2  14C10 epitope spans into EDIII from neighboring dimer. The 14C10 epitope sits primarily over EDI of a single envelope dimer, but it interacts with additional residues of EDIII in the adjacent envelope dimer. Download FIG S2, PDF file, 0.4 MB. Copyright © 2017 Gallichotte et al. 2017 Gallichotte et al. This content is distributed under the terms of the Creative Commons Attribution 4.0 International license.

## DISCUSSION

While a number of reverse genetic systems for dengue viruses have been developed, significant obstacles, including replication attenuation, sequence alteration, as well as labor- and cost-intensive techniques, have limited their utility (6). In this study, we further characterize a novel DENV1 molecular clone platform that is patterned after coronavirus systems and describe additional details, including transcript infectivity and recombinant virus phenotypes *in vitro* (19, 28–31). Generating a panel of contiguous cDNAs that span the entire genome, the divided DENV1 reverse genetic system overcomes toxic elements within itself and allows propagation in bacteria. Importantly, directional assembly and *in vitro* transcription allow electroporation of full-length infectious RNA that directly corresponds to the wild-type virus sequence. As a result, the virus derived from the infectious clone maintains similar replication in multiple cell types (Fig. 1E to G) as well as complete fidelity in regard to antibody binding and virus neutralization (Fig. 2 and 3). Building on previous epitope swap mutants (19, 32), we employed this reverse genetic system to generate a DENV1 viral mutant that displayed a known monoclonal antibody epitope from DENV4, gaining 5H2 monoclonal antibody binding and neutralization to the donor sequence strain. However, the mutant virus also disrupted binding and neutralization of two DENV1-specific monoclonal antibodies despite retaining the majority of their targeted antigenic residues (Table 1 and Fig. 5). While other DENV1-specific monoclonal antibodies maintain effective binding and neutralization (Fig. 5C), the data highlight the opportunity cost of domain swaps even in the context of partial epitope disruption. Overall, the results also illustrate the utility of this newly developed infectious clone systems as a platform to study DENV1 infection, pathogenesis, and immunity.

For the DENV vaccine field, this reverse genetic system amplifies opportunities in virus design that are already being explored. Previous work by our lab has utilized the DENV2 and DENV4 infectious clones to define and transfer a critical antibody binding epitope in DENV2 identified by structural analysis (22, 32). In this case, we described a recombinant DENV4 virus that displayed a heterologous DENV2 epitope, while preserving the major neutralizing epitopes on DENV4 (32). In addition, previous work using this DENV1 infectious clone was able to transfer part of the DENV3 MAb 5J7 epitope, resulting in a partial gain of binding and neutralization by 5J7 but no loss of binding to DENV1 MAb 1F4 (19). Importantly, both the DENV2 2D22 and DENV3 5J7 MAb epitopes are distinct from the 1F4 and 14C10 epitopes transplanted here. Coupled with data from the current study, the approach to disrupt epitopes within the context of live virus highlights an independent and powerful approach to quickly validate structural predictions of key residues. Importantly, full characterization of these viral epitopes and their portability between DENV serotypes open new approaches to vaccine development. This work defines a rationally designed chimeric virus that uses MAb-envelope structural interactions to identify residues associated with the DENV4 MAb 5H2 epitope that were sufficient for the gain of binding and neutralization. These changes lead to the disruption of multiple overlapping DENV1 epitopes, highlighting a potential problem that must be addressed in DENV vaccine design. Interestingly, despite maintaining nearly two-thirds of the DENV1 1F4 epitope in the chimeric virus, binding and neutralization were almost completely lost, indicating that the critical residue determinants for antibody interactions were among those that were changed. Another DENV1 MAb, 14C10, which maintained a much larger percentage of its epitope in the chimeric virus, lost a smaller amount of binding yet still lost nearly complete neutralization by 14C10. This suggests that while the remaining epitope can still be partially bound by 14C10, the interaction is insufficient to fully neutralize the virus. Structure-guided design could identify additional residues to further expand the transplanted DENV4 epitope to fully ablate DENV1 MAb and polyclonal binding and neutralization. Together, these data illustrate the utility of mapping epitopes using a combination of structure and reveres genetics. With a similar approach employed for human norovirus (33–37), these resources can be applied to generate diagnostic reagents and to create a map of epitopes ideal for designing chimeric bivalent vaccines that produce broad-spectrum neutralization, a critical issue in DENV pathogenesis.

Moving beyond vaccine immunity, the infectious clone system provides opportunities to contribute to other areas of DENV research. Exploring virus-host interactions has been complicated by attenuation of traditional clone-derived virus (6). Our system maintains replication equivalent to that of the wild-type strain, allowing examination of the impact of specific point mutations or viral protein deletion without the complication of interpreting baseline clone attenuation. In addition to these areas, the infectious clone system may be a useful resource in identifying virulence determinants, host specificity, viral protein function, and RNA structural elements, as well as testing of antiviral therapeutics.

Overall, the reverse genetic system characterized in this study represents a major resource for the study of DENV1 infection and pathogenesis. Building on previous reports (19), this clone system provides several important advantages to study DENV1, including robust propagation in bacteria, fidelity to wild-type sequence, and absence of replication attenuation. Importantly, both antibody binding and virus neutralization indicate uniform antigenicity with the wild-type strain permitting epitope mapping. Coupled with structural studies, the reverse genetic system can be manipulated to produce chimeric vaccines harboring conserved epitopes from multiple serotypes. While it is unlikely that neutralizing epitopes from all four serotypes could be combined into a single viable chimeric DENV virus, it is possible that two bivalent viruses that contain neutralizing epitopes from all four serotypes (e.g., rDENV1/3 and rDENV4/2) could be generated. This advance offers a promising strategy to produce broad protection against the DENV family.

## MATERIALS AND METHODS

### Virus construction.

Recombinant DENV1 West Pac ’74 infectious clone (rDENV1ic) was constructed using a four-cDNA cloning strategy as previously described (19, 32, 38). Briefly, the DENV1 genome was divided into four fragments and subcloned into separate cDNA plasmids with unique type IIS restriction endonuclease cleavage sites at the 5′ and 3′ ends of each fragment. A PflMI site was removed using standard molecular techniques. A T7 promoter was introduced into the 5′ end of the plasmid A fragment, and plasmid DNA was grown in *Escherichia coli* cells. Purified plasmid DNA was enzyme digested, purified, and ligated together with T4 DNA ligase. Infectious genome-length capped viral RNA transcripts were generated with T7 polymerase. RNA was electroporated into C6/36 cells and incubated 4 to 6 days. Cell culture supernatant containing virus was harvested, centrifuged at maximum speed to remove cellular debris, and passaged onto C6/36 cells to generate a passage one virus stock.

### rDENV1ic-EDI mutant construction.

For mutant virus generation, 12 predicted contact residues of 5H2 (24, 25) were identified that differ between DENV1 and DENV4 (Table 1). rDENV1ic plasmid A fragment was redesigned to encode these 12-amino-acid changes in the envelope protein to create rDENV1ic-EDI. The new plasmid A was digested and ligated with WT rDENV1ic plasmids B, C, and D as described above to generate the rDENV1ic-EDI mutant virus.

### Cells.

Cells were cultured as previously described by our group (19, 32). C6/36 cells were grown in Gibco minimal essential medium (MEM) at 32°C. DC-SIGN-expressing U937 cells (U937+DC-SIGN) were maintained in RPMI 1640 at 37°C. Media were supplemented with fetal bovine serum (FBS) (5% for C6/36 and U937+DC-SIGN cells) which was lowered to 2% after infection. C6/36 and U937+DC-SIGN media were supplemented with nonessential amino acids, and U937+DC-SIGN medium were also supplemented with l-glutamine and 2-mercaptoethanol. All media were additionally supplemented with 100 U/ml penicillin, 100 μg/ml streptomycin, and 0.25 μg/ml amphotericin B. All cells were incubated in 5% CO_2_.

### Virus titration and immunostaining.

Cell culture plates (24-well plates) were seeded with C6/36 cells to be confluent at the time of infection. The cell growth media were removed, and virus stocks were serially diluted 10-fold and then added to cells for 1 h at 32°C (C6/36) with gentle rocking. After incubation, cells were overlaid with 1% methylcellulose in Opti-MEM I (Gibco) supplemented with 2% FBS, nonessential amino acids, 100 U/ml penicillin, 100 μg/ml streptomycin, and 0.25 μg/ml amphotericin B and incubated at 32°C. After 4 to 6 days of incubation, the overlay was removed, and the cells were washed with phosphate-buffered saline (PBS) and fixed in 80% methanol. The cells were blocked in 5% nonfat dried milk in PBS (blocking buffer) and then incubated for 1 h at 37°C with anti-prM MAb 2H2 and anti-E MAbs 4G2 and DV1-E103 diluted in blocking buffer. The cells were washed two times with PBS and then incubated for 1 h at 37°C with horseradish peroxidase (HRP)-conjugated goat anti-mouse antibody (Sigma) diluted in blocking buffer. The plates were washed two times with PBS, and foci were developed using TrueBlue HRP substrate (KPL).

### Infectious center assay.

C6/36 cells electroporated with rDENV1ic RNA were diluted in Opti-MEM I (Gibco), added to a confluent monolayer of C6/36 cells, overlaid with 1% methylcellulose, and incubated for 4 days. After incubation, cells were fixed and stained as described above. The percentage of electroporated cells capable of making viable infectious virus was calculated as follows: (number of foci/number of electroporated cells plated) × 100. 

### Growth curves.

To determine the amount of virus cells can produce, C6/36 cells were inoculated at a multiplicity of infection (MOI) of 0.01. Every 24 h, all cell culture supernatant was harvested (volume was replaced with fresh medium) and frozen at −80°C. The titers of the virus samples were determined as described above. To determine the kinetics at which cells become infected, C6/36 cells were inoculated at an MOI of 0.01. Every 12 h, cell culture medium was removed, and the cells were washed with PBS, fixed, permeabilized, and probed with anti-prM MAb 2H2 conjugated to Alexa Fluor 488. Infected cells were quantified using a Guava flow cytometer (Millipore). U937+DC-SIGN cells were infected at an initial infection of 2%, and every 12 h, a sample of cells were harvested, fixed, and stained as described for C6/36 cells.

### Binding enzyme-linked immunosorbent assay.

The plates were coated with either 100 ng/well each mouse MAb 4G2 and 2H2 or 200 ng/well human MAb 1C19 overnight at 4°C. The plates were washed with Tris-buffered saline with 0.05% Tween (TBST) and blocked in 3% nonfat dried milk in TBST (blocking buffer), and equal quantities of virus (as previously titrated by enzyme-linked immunosorbent assay [ELISA]) were added and incubated for 1 h at 37°C. The plates were washed, and primary human MAbs were diluted fourfold in blocking buffer and added to the plates for 1 h. The plates were washed, and alkaline phosphate (AP)-conjugated secondary antibodies were added for 1 h at 37°C. The plates were washed and developed using *p*-nitrophenyl phosphate substrate, and color changes were quantified by spectrophotometry as previously described (19, 32).

### Neutralization assays.

For the focus reduction neutralization test (FRNT), 24-well cell culture plates were seeded with C6/36 cells to be confluent at time of infection. MAbs were diluted fourfold, mixed with ~45 focus-forming units (FFU) of virus, and incubated for 1 h at 32°C. After incubation, the virus-MAb mixture was added to C6/36 cells for 1 h at 32°C with gentle rocking. The overlay was added, and the cells were incubated for 4 to 6 days. The cells were fixed and stained as described above. For the flow-based neutralization assay, MAbs were diluted fourfold, mixed with virus (previously titrated to equal ~15% infection with no MAb present), and incubated for 1 h at 37°C. After incubation, the virus-MAb mixture was added to 5 × 10^4^ U937+DC-SIGN cells for 2 h at 37°C. The cells were then pelleted, washed two times with fresh medium, and then incubated for 24 h. After incubation, the cells were fixed, permeabilized, and stained as described above for U937+DC-SIGN growth curves.

### Accession number(s).

Wild-type DENV1 and DENV4 sequences were derived from GenBank accession numbers U88535.1 and KJ160504.1. Monoclonal antibodies were synthesized from their deposited Protein Data Bank sequences (PDB identifiers [ID] shown in brackets) (MAb 1F4 [4C2I], 14C10 [4CAU], 2D22 4UIF], 5J7 [3J6U], 5H2 [3UAJ]).

## References

[1] Bhatt S, Gething PW, Brady OJ, Messina JP, Farlow AW, Moyes CL, Drake JM, Brownstein JS, Hoen AG, Sankoh O, Myers MF, George DB, Jaenisch T, Wint GR, Simmons CP, Scott TW, Farrar JJ, Hay SI 2013 The global distribution and burden of dengue. Nature 496:504–507. doi:10.1038/nature12060. 23563266 PMC3651993

[2] Guzman MG, Gubler DJ, Izquierdo A, Martinez E, Halstead SB 2016 Dengue infection. Nat Rev 2:16055. doi:10.1038/nrdp.2016.55. 27534439

[3] Diamond MS, Pierson TC 2015 Molecular insight into dengue virus pathogenesis and its implications for disease control. Cell 162:488–492. doi:10.1016/j.cell.2015.07.005. 26232221 PMC4522276

[4] Halstead SB 2007 Dengue. Lancet 370:1644–1652. doi:10.1016/S0140-6736(07)61687-0. 17993365

[5] Khetarpal N, Khanna I 2016 Dengue fever: causes, complications, and vaccine strategies. J Immunol Res 2016:6803098. doi:10.1155/2016/6803098. PMC4971387 27525287

[6] Aubry F, Nougairède A, Gould EA, de Lamballerie X 2015 Flavivirus reverse genetic systems, construction techniques and applications: a historical perspective. Antiviral Res 114:67–85. doi:10.1016/j.antiviral.2014.12.007. 25512228 PMC7173292

[7] Blaney JE Jr, Durbin AP, Murphy BR, Whitehead SS 2006 Development of a live attenuated dengue virus vaccine using reverse genetics. Viral Immunol 19:10–32. doi:10.1089/vim.2006.19.10. 16553547

[8] Mukherjee S, Pierson TC, Dowd KA 2014 Pseudo-infectious reporter virus particles for measuring antibody-mediated neutralization and enhancement of dengue virus infection. Methods Mol Biol 1138:75–97. doi:10.1007/978-1-4939-0348-1_6. 24696332

[9] Shang W, Liu J, Yang J, Hu Z, Rao X 2012 Dengue virus-like particles: construction and application. Appl Microbiol Biotechnol 94:39–46. doi:10.1007/s00253-012-3958-7. 22382168

[10] Coffey LL, Vignuzzi M 2011 Host alternation of Chikungunya virus increases fitness while restricting population diversity and adaptability to novel selective pressures. J Virol 85:1025–1035. doi:10.1128/JVI.01918-10. 21047966 PMC3020036

[11] Pierro DJ, Salazar MI, Beaty BJ, Olson KE 2006 Infectious clone construction of dengue virus type 2, strain Jamaican 1409, and characterization of a conditional E6 mutation. J Gen Virol 87:2263–2268. doi:10.1099/vir.0.81958-0. 16847122

[12] Kinney RM, Butrapet S, Chang GJ, Tsuchiya KR, Roehrig JT, Bhamarapravati N, Gubler DJ 1997 Construction of infectious cDNA clones for dengue 2 virus: strain 16681 and its attenuated vaccine derivative, strain PDK-53. Virology 230:300–308. doi:10.1006/viro.1997.8500. 9143286

[13] Suzuki R, de Borba L, Duarte dos Santos CN, Mason PW 2007 Construction of an infectious cDNA clone for a Brazilian prototype strain of dengue virus type 1: characterization of a temperature-sensitive mutation in NS1. Virology 362:374–383. doi:10.1016/j.virol.2006.11.026. 17289102 PMC2396755

[14] Usme-Ciro JA, Lopera JA, Enjuanes L, Almazán F, Gallego-Gomez JC 2014 Development of a novel DNA-launched dengue virus type 2 infectious clone assembled in a bacterial artificial chromosome. Virus Res 180:12–22. doi:10.1016/j.virusres.2013.12.001. 24342140 PMC7114509

[15] Polo S, Ketner G, Levis R, Falgout B 1997 Infectious RNA transcripts from full-length dengue virus type 2 cDNA clones made in yeast. J Virol 71:5366–5374. 9188607 10.1128/jvi.71.7.5366-5374.1997 PMC191775

[16] Pu SY, Wu RH, Yang CC, Jao TM, Tsai MH, Wang JC, Lin HM, Chao YS, Yueh A 2011 Successful propagation of flavivirus infectious cDNAs by a novel method to reduce the cryptic bacterial promoter activity of virus genomes. J Virol 85:2927–2941. doi:10.1128/JVI.01986-10. 21228244 PMC3067970

[17] Siridechadilok B, Gomutsukhavadee M, Sawaengpol T, Sangiambut S, Puttikhunt C, Chin-inmanu K, Suriyaphol P, Malasit P, Screaton G, Mongkolsapaya J 2013 A simplified positive-sense-RNA virus construction approach that enhances analysis throughput. J Virol 87:12667–12674. doi:10.1128/JVI.02261-13. 24049164 PMC3838137

[18] Aubry F, Nougairède A, de Fabritus L, Querat G, Gould EA, de Lamballerie X 2014 Single-stranded positive-sense RNA viruses generated in days using infectious subgenomic amplicons. J Gen Virol 95:2462–2467. doi:10.1099/vir.0.068023-0. 25053561 PMC4202267

[19] Messer WB, Yount BL, Royal SR, de Alwis R, Widman DG, Smith SA, Crowe JE Jr, Pfaff JM, Kahle KM, Doranz BJ, Ibarra KD, Harris E, de Silva AM, Baric RS 2016 Functional transplant of a dengue virus serotype 3 (DENV3)-specific human monoclonal antibody epitope into DENV1. J Virol 90:5090–5097. doi:10.1128/JVI.00155-16. 26962223 PMC4859728

[20] de Alwis R, Smith SA, Olivarez NP, Messer WB, Huynh JP, Wahala WM, White LJ, Diamond MS, Baric RS, Crowe JE Jr, de Silva AM 2012 Identification of human neutralizing antibodies that bind to complex epitopes on dengue virions. Proc Natl Acad Sci U S A 109:7439–7444. doi:10.1073/pnas.1200566109. 22499787 PMC3358852

[21] Fibriansah G, Tan JL, Smith SA, de Alwis AR, Ng TS, Kostyuchenko VA, Ibarra KD, Wang J, Harris E, de Silva A, Crowe JE Jr, Lok SM 2014 A potent anti-dengue human antibody preferentially recognizes the conformation of E protein monomers assembled on the virus surface. EMBO Mol Med 6:358–371. doi:10.1002/emmm.201303404. 24421336 PMC3958310

[22] Fibriansah G, Ibarra KD, Ng TS, Smith SA, Tan JL, Lim XN, Ooi JS, Kostyuchenko VA, Wang J, de Silva AM, Harris E, Crowe JE Jr, Lok SM 2015 Dengue virus. Cryo-EM structure of an antibody that neutralizes dengue virus type 2 by locking E protein dimers. Science 349:88–91. doi:10.1126/science.aaa8651. 26138979 PMC4672004

[23] Fibriansah G, Tan JL, Smith SA, de Alwis R, Ng TS, Kostyuchenko VA, Jadi RS, Kukkaro P, de Silva AM, Crowe JE, Lok SM 2015 A highly potent human antibody neutralizes dengue virus serotype 3 by binding across three surface proteins. Nat Commun 6:6341. doi:10.1038/ncomms7341. 25698059 PMC4346626

[24] Lai CJ, Goncalvez AP, Men R, Wernly C, Donau O, Engle RE, Purcell RH 2007 Epitope determinants of a chimpanzee dengue virus type 4 (DENV-4)-neutralizing antibody and protection against DENV-4 challenge in mice and rhesus monkeys by passively transferred humanized antibody. J Virol 81:12766–12774. doi:10.1128/JVI.01420-07. 17881450 PMC2169078

[25] Cockburn JJ, Navarro Sanchez ME, Goncalvez AP, Zaitseva E, Stura EA, Kikuti CM, Duquerroy S, Dussart P, Chernomordik LV, Lai CJ, Rey FA 2012 Structural insights into the neutralization mechanism of a higher primate antibody against dengue virus. EMBO J 31:767–779. doi:10.1038/emboj.2011.439. 22139356 PMC3273384

[26] Teoh EP, Kukkaro P, Teo EW, Lim AP, Tan TT, Yip A, Schul W, Aung M, Kostyuchenko VA, Leo YS, Chan SH, Smith KG, Chan AH, Zou G, Ooi EE, Kemeny DM, Tan GK, Ng JK, Ng ML, Alonso S, Fisher D, Shi PY, Hanson BJ, Lok SM, MacAry PA 2012 The structural basis for serotype-specific neutralization of dengue virus by a human antibody. Sci Transl Med 4:139ra83. doi:10.1126/scitranslmed.3003888. 22723463

[27] Shrestha B, Brien JD, Sukupolvi-Petty S, Austin SK, Edeling MA, Kim T, O’Brien KM, Nelson CA, Johnson S, Fremont DH, Diamond MS 2010 The development of therapeutic antibodies that neutralize homologous and heterologous genotypes of dengue virus type 1. PLoS Pathog 6:e1000823. doi:10.1371/journal.ppat.1000823. 20369024 PMC2848552

[28] Yount B, Curtis KM, Fritz EA, Hensley LE, Jahrling PB, Prentice E, Denison MR, Geisbert TW, Baric RS 2003 Reverse genetics with a full-length infectious cDNA of severe acute respiratory syndrome coronavirus. Proc Natl Acad Sci U S A 100:12995–13000. doi:10.1073/pnas.1735582100. 14569023 PMC240733

[29] Yount B, Curtis KM, Baric RS 2000 Strategy for systematic assembly of large RNA and DNA genomes: transmissible gastroenteritis virus model. J Virol 74:10600–10611. doi:10.1128/JVI.74.22.10600-10611.2000. 11044104 PMC110934

[30] Donaldson EF, Yount B, Sims AC, Burkett S, Pickles RJ, Baric RS 2008 Systematic assembly of a full-length infectious clone of human coronavirus NL63. J Virol 82:11948–11957. doi:10.1128/JVI.01804-08. 18818320 PMC2583659

[31] Scobey T, Yount BL, Sims AC, Donaldson EF, Agnihothram SS, Menachery VD, Graham RL, Swanstrom J, Bove PF, Kim JD, Grego S, Randell SH, Baric RS 2013 Reverse genetics with a full-length infectious cDNA of the Middle East respiratory syndrome coronavirus. Proc Natl Acad Sci U S A 110:16157–16162. doi:10.1073/pnas.1311542110. 24043791 PMC3791741

[32] Gallichotte EN, Widman DG, Yount BL, Wahala WM, Durbin A, Whitehead S, Sariol CA, Crowe JE Jr, de Silva AM, Baric RS 2015 A new quaternary structure epitope on dengue virus serotype 2 is the target of durable type-specific neutralizing antibodies. mBio 6(5):e01461-15. doi:10.1128/mBio.01461-15. 26463165 PMC4620467

[33] Debbink K, Donaldson EF, Lindesmith LC, Baric RS 2012 Genetic mapping of a highly variable norovirus GII.4 blockade epitope: potential role in escape from human herd immunity. J Virol 86:1214–1226. doi:10.1128/JVI.06189-11. 22090110 PMC3255819

[34] Debbink K, Lindesmith LC, Donaldson EF, Swanstrom J, Baric RS 2014 Chimeric GII.4 norovirus virus-like-particle-based vaccines induce broadly blocking immune responses. J Virol 88:7256–7266. doi:10.1128/JVI.00785-14. 24741081 PMC4054422

[35] Debbink K, Lindesmith LC, Ferris MT, Swanstrom J, Beltramello M, Corti D, Lanzavecchia A, Baric RS 2014 Within-host evolution results in antigenically distinct GII.4 noroviruses. J Virol 88:7244–7255. doi:10.1128/JVI.00203-14. 24648459 PMC4054459

[36] Lindesmith LC, Costantini V, Swanstrom J, Debbink K, Donaldson EF, Vinjé J, Baric RS 2013 Emergence of a norovirus GII.4 strain correlates with changes in evolving blockade epitopes. J Virol 87:2803–2813. doi:10.1128/JVI.03106-12. 23269783 PMC3571402

[37] Lindesmith LC, Donaldson E, Leon J, Moe CL, Frelinger JA, Johnston RE, Weber DJ, Baric RS 2010 Heterotypic humoral and cellular immune responses following Norwalk virus infection. J Virol 84:1800–1815. doi:10.1128/JVI.02179-09. 20007270 PMC2812379

[38] Messer WB, Yount B, Hacker KE, Donaldson EF, Huynh JP, de Silva AM, Baric RS 2012 Development and characterization of a reverse genetic system for studying dengue virus serotype 3 strain variation and neutralization. PLoS Neglect Trop Dis 6:e1486. doi:10.1371/journal.pntd.0001486. PMC3289595 22389731

