# Supplementary material for: Epitope Addition and Ablation via Manipulation of a Dengue Virus Serotype 1 Infectious Clone
Source: mSphere. 2017 Feb 22;2(1):e00380-16. doi: 10.1128/mSphere.00380-16 (PMC5322348; doi:10.1128/mSphere.00380-16)
Supplement: TABLE S1 [file sph002172240st3.pdf]

Table S1

Serotype-Specificity	MAb	Species	Epitope
**DENV1**	1F4	Human	EDI, EDI/II hinge
	14C10	Human	EDI, EDI/II hinge
	1C19.2	Human	EDIII
	E103	Mouse	EDIII
**DENV2**	2D22	Human	EDIII/EDII
**DENV3**	5J7	Human	EDI/II hinge
**DENV4**	5H2	Human	EDI
