# Supplementary figures and images for: Epitope Addition and Ablation via Manipulation of a Dengue Virus Serotype 1 Infectious Clone
Source: mSphere. 2017 Feb 22;2(1):e00380-16. doi: 10.1128/mSphere.00380-16 (PMC5322348; doi:10.1128/mSphere.00380-16)

**Fig. S1**

**A**

**1F4 Neut**

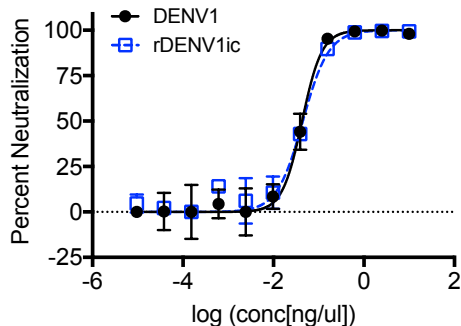

**B**

**14C10 Neut**

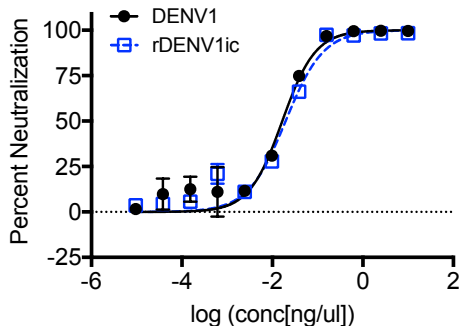

**C**

**1C19.2 Neut**

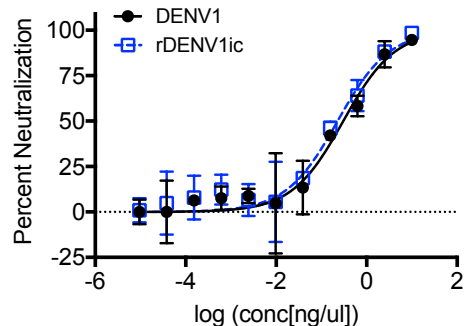

**D**

**2D22 Neut**

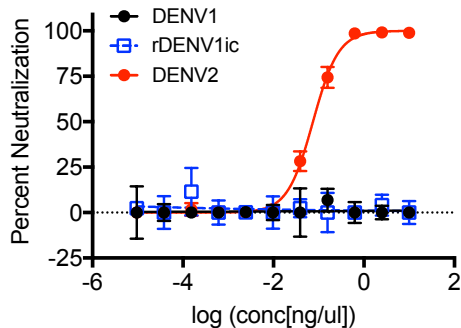

**E**

**5J7 Neut**

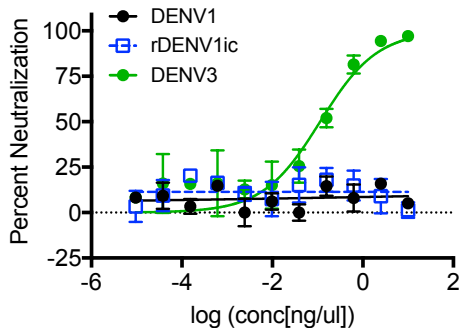

**F**

**5H2 Neut**

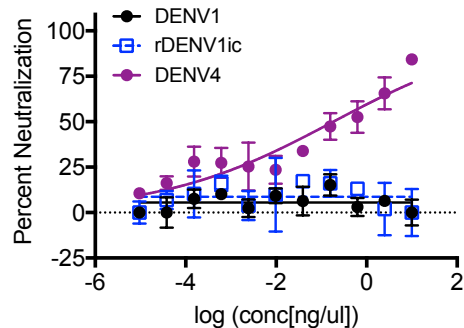

Supplement: FIG S1 [file sph002172240sf1.pdf]

**Fig. S2**

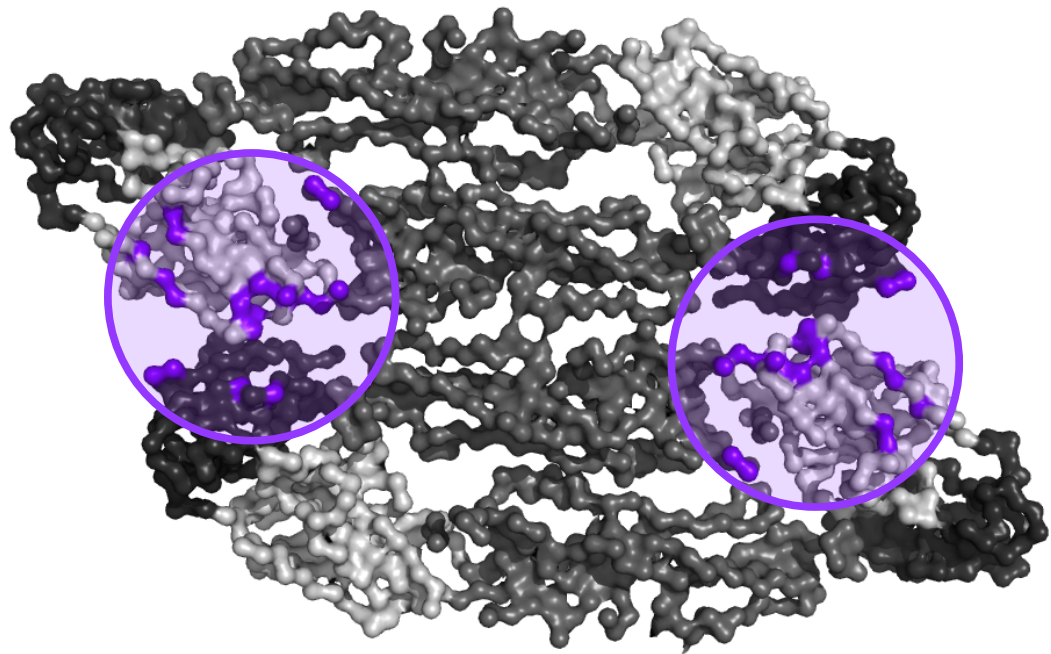

Supplement: FIG S2 [file sph002172240sf2.pdf]
